# Is there a difference between the effects of phytoestrogens and non-phytoestrogens medicinal plants on sexual health? A systematic review and meta-analysis

**DOI:** 10.18502/ijrm.v21i11.14652

**Published:** 2023-12-19

**Authors:** Masoumeh Sayahi, Afsaneh Keramat, Firoozeh Nourimand1 Ph.D. Candidate, Hakimeh Mohammadzadeh

**Affiliations:** ^1^Student Research Committee, School of Nursing and Midwifery, Shahroud University of Medical Sciences, Shahroud, Iran.; ^2^Center for Health Related Social and Behavioral Sciences Research, Shahroud University of Medical Sciences, Shahroud, Iran.; ^3^Department of Nursing, Shoushtar Faculty of Medical Sciences, Shoushtar, Iran.

**Keywords:** Medicinal, Plants, Sexual health, Women, Meta-analysis.

## Abstract

**Background:** The quality of life of a person and her sexual partner is impacted by sexual function. Sexual function disorders have a significant prevalence in society. There are different treatments for sexual disorders, including herbal therapies. **Objective:** This study aimed to do a comparison of phytoestrogens and non-phytoestrogens medicinal plant's effects on sexual health in pre- and postmenopausal women. **Materials and Methods:** This systematic review and meta-analysis was performed to identify relevant articles in electronic databases such as Web of Science, Scopus, PubMed, Cochrane Library, Google Scholar, and SID for English- and Persian-language articles published up to December 2021. The Cochrane collaboration tool was used to assess the risk of bias. Heterogeneity was assessed using I^2^ statistics. **Results:** Of 5428 records retrieved by searching the databases, after removing duplicate and irrelevant articles, 39 articles were included based on the inclusion criteria in the study. Finally, 18 articles with 1299 participants were included in the meta-analysis. 18 randomized clinical trial studies of phytoestrogens (n = 13) and non-phytoestrogens (n = 5) plants that used the female sexual function index questionnaire and reported the mean difference (MD) and standard deviation were included in the meta-analysis. The effects of phytoestrogens and non-phytoestrogens plants on the sexual health of postmenopausal women appear to differ significantly from one another, according to the meta-analysis (MD = 7.59; 95% CI = 4.56-10.60 and MD = 3.19; 95% CI = 1.25-5.13, respectively) but this difference was not observed in premenopausal women. **Conclusion:** The effect of phytoestrogens plants is more in menopausal women, and they can be advised to use these herbs.

## 1. Introduction

The inability to have healthy emotional relationships, the inability for society to flourish, infanticide, and even death will result from a lack of sexual health and security. These emotions include anger and frustration, depression, drug abuse, and parents' diminished physical and mental capacity for parenting and child care (1).

Sexual health, according to the definition of the World Health Organization, is the state of physical, mental and social health of a person in a sexual relationship (2, 3). Humans have always focused their attention, curiosity, and interest on sexual function, which is the body's response to various stages of the sexual response cycle (4). One of the crucial aspects of a woman's health is her sexual function, which impacts her sense of well-being, welfare, and quality of life. Thus, any issues with sexual function may severely impact her quality of life (5).

Based on the $10^{\text{th}}$ revision of the International Statistical Classification of Diseases and Related Health Problems, sexual dysfunction is the inability to engage in desirable intercourse. Sexual arousal, desire, orgasm, and genital pelvic pain/penetration disorder are just a few examples of the various stages of the sexual cycle in which women might have sexual dysfunction (6). According to the latest edition of the Diagnostic and Statistical Manual 5, sexual disorders are “a heterogeneous group of disorders that are usually characterized by a clinically significant impairment in a person's ability to respond sexually or experience sexual pleasure". Thus, “female sexual dysfunction" is a collective term for 4 different disorders identified in the diagnostic and statistical manual 5: female orgasmic disorder, genito-pelvic pain/penetration disorder, drug-induced sexual dysfunction and female sexual interest/arousal disorder (7). Due to the taboo nature of this issue, the prevalence of sexual function abnormalities are underreported between 30 and 50% (8). 38% of married women, aged between 19 and 49 yr, who participated in a poll of the local population in Hong Kong reported having some form of sexual dysfunction. Age-related increases in the disorder's prevalence have been recorded (9).

As per the female sexual function index (FSFI), the prevalence of diminished libido, lack of sexual excitement, and absence of orgasm in Iranian women aged between 20 and 60 yr were 35, 30, 37, and 26%, respectively (10). Several techniques have been developed to assess women's sexual function. The FSFI measure has been the one most widely employed, a 19-item scale with 6 domains: desire, arousal, lubrication, orgasm, pain, and pleasure (7). Many treatments have been investigated to improve this disorder. Despite the increase in access to effective modern medical treatments, the prevalence of side effects associated with these drugs has led to the emergence of complementary medicine. Medicinal herbs, acupuncture, and massage are some of these treatments (11).

The comparison of phytoestrogens and non-phytoestrogen plants' effects on sexual function in women was the goal of this systematic review and meta-analysis study.

## 2. Materials and Methods 

### Literature and search strategy

This systematic review and meta-analysis was performed based on reporting items for systematic reviews and meta-analysis according to PRISMA guidelines. 2 authors (Masoumeh Sayahi and Afsaneh Keramat) conducted a systematic literature search in Web of Science, Scopus, PubMed, Cochrane Library, and Google Scholar, SID for English- and Persian-language articles published up to December 2021 using the English or Persian keywords listed in table I in titles/abstracts.

**Table 1:** Search term


**Block 1**	Medicinal plants, herbal medicines, herbal, herbal supplements
**Block 2**	“sexual dysfunction of women" OR “sexual dysfunction of reproductive age women" OR “sexual dysfunction of menopausal women" OR “sexual health" OR “orgasmic disorder" OR “sexual satisfaction" OR “female sexual dysfunction" OR “sexual dysfunction"
**Block 3**	“Randomized control trial" OR “RCT" OR “randomized trial"

### Inclusion criteria

The patient, intervention, comparison, and outcome framework was used to identify components of clinical evidence. Randomized controlled trial studies with the following characteristics for which full text was available were included in this systematic review and meta-analysis:

P- The target population was women with sexual dysfunction (and do not have a chronic disease).

I- The intervention was phytoestrogen or non-phytoestrogen medicinal plants.

C- The comparison group was women with sexual dysfunction who received placebo.

O- The outcome included improved sexual health.

### Exclusion criteria

Some studies were excluded for the following reasons: absence of keywords in the title of the article, incorrect comparison subjects and study design, studies with non-extractable data, studies with a high risk of bias based on Cochrane Collaboration tool.

### The criteria for selecting herbs 

Phytoestrogens and non-phytoestrogens medicinal plants that are effective on sexual health, and eligible articles that researched the effects of these herbs on women's sexual health.

### Screening and data collection 

All search results for title page, abstract and full text were imported into EndnoteX7. 2 reviewers selected studies in 2 separate rounds (M.S and A.K). After screening, the extracted data were independently entered into an online Google spreadsheet by 2 reviewers (M.S and A.K) in a standardized order: author name, year of publication, year of study, country or region studied, design, sample size, population characteristics, study settings, preventive outcomes, and assessment tools. The overall agreement between the reviewers was 95%, and all disagreements were discussed and analyzed by examining the inclusion or exclusion criteria and achieving consensus. Finally, another staff member (FN) verified the entire process.

### Quality assessment and risk of bias

**Figure 1:**
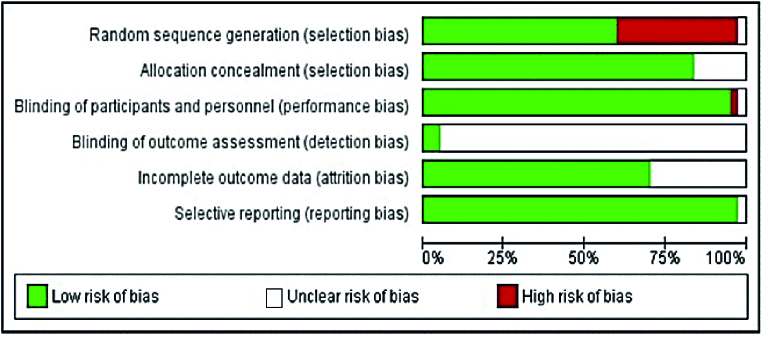
Risk of bias assessment of clinical trial articles used in the study.

**Figure 2:**
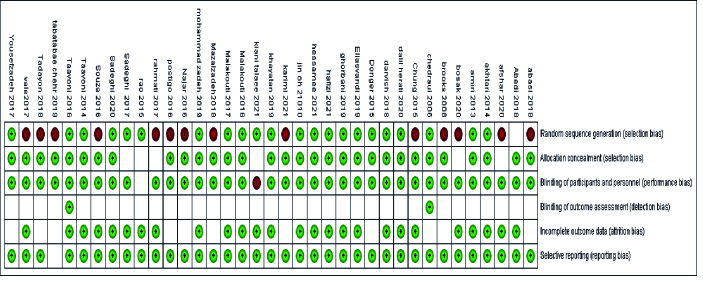
Risk of bias for each study. The green color indicates low risk of bias, white indicates unclear risk of bias, and red indicates high risk of bias.

The Cochrane Collaboration tool was used to assess the risk of bias for randomized controlled by Review Manager 5.1 software. Bias was assessed as a result for 6 areas of bias including random sequence generation (selection bias), blinding of participants (performance bias), allocation concealment (selection bias), blinding of outcome assessment (detection bias), incomplete outcome data (attrition bias), and selective reporting (reporting bias) (3). The results are shown in figure 1 as low, high, and unclear risks. The risk of bias for each study is shown in figure 2.

### Data analysis and description of findings

All effect sizes for sexual health were calculated using mean differences and standard deviations. Heterogeneity was evaluated using the I^2^ statistic to determine the presence of real or random differences in the study results. I^2^ is the degree of variance change for the calculated effect size, which results from heterogeneity in studies. I^2^ values include 0%, 25%, 50%, and 75%, indicating no, low, medium, and high heterogeneity, respectively (3). P ≥ 0.05 was considered significant to evaluate heterogeneity. Heterogeneity was considered statistically significant (p < 0.05 or I^2^ > 50%) and the random effects model was used. Based on the obtained results, a meta-analysis was performed. And articles were included in the meta-analysis in which sexual health was measured based on the FSFI questionnaire and the reason for that was increased accuracy. The calculations were performed using the Review Manager 5.1 software.

## 3. Results

### Study selection and characteristics

**Figure 3:**
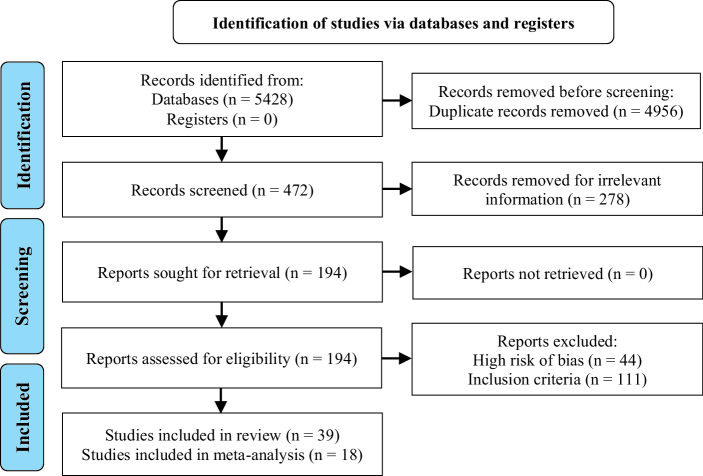
PRISMA flow diagram.

According to the above search term (Table I), the systematic electronic literature search yielded 5428 entries. After removing duplication, 472 articles remained. Of the remaining articles, 433 articles were excluded for the following reasons: 278 articles due to irrelevant information, 44 articles due to high risk of bias and 111 articles due to lack of inclusion criteria. Ultimately, we considered 39 articles based on the inclusion criteria (with 2816 participants), and 18 articles with 1299 participants were included in the meta-analysis. Figure 3 shows the PRISMA flow chart that illustrates the systematic process used to conduct the review. Medicinal plants were divided into 2 categories:

1) Phytoestrogen plants: Vitex (1 article), red clover (2), squill oil (2), *Trigonella foenum *(*T. foenum*) (2), fennel (2), *Punica granatum L* (*P. granatum L*) (1), combination of oak, *P. granatum L* and *T. foenum* seeds (1), ginkgo biloba (*G. biloba*) (4), maca (1), red ginseng (*Panax ginseng, P. ginseng*) (3), celery seed (1), carrot seeds (1), chamomile (1), combined herbal capsule (*Terminalia chebula*, fresh green raisins, senna leaves, tangerine, carnation, anise, anison, golqand, and fresh green raisins) (1).

2) Non-phytoestrogen plants: alcea (1 article), lemon balm (1), combination of *Melissa officinalis *(*M. officinalis*), fennel extract, and *Nigella sativa *(1), ashwagandha (1), date palm pollen (2), saffron (2), zygophyllaceae (5), aphrodite (zygophyllaceae, ginger, saffron, and cinnamon) (2).

18 studies were similar in terms of the questionnaire (which examined sexual function with the FSFI and reported the mean and standard deviation) and were included in the meta-analysis. These studies were among 39 randomized control trial articles that investigated the effects of herbal medicines (phytoestrogens and non-phytoestrogen plants) on the sexual health of pre- and postmenopausal women. The summary of the studies is given in table II and III.

### Classification and description studies

#### Phytoestrogens plants 

Flavonoids, coumestans, and lignans, which have the potential to hold the estrogen receptors alpha and beta together, are the 3 prominent families of phytoestrogens (12).

#### 3.2.1.1. Vitex-agnus castus

The Vitex plant belongs to the category of phytoestrogens. The chemical compounds of the Vitex plant are flavonoids, glycosides, saponins, and fatty acids such as linoleic acid. The impact of the Vitex plant on the sexual dysfunction of women of reproductive age was examined. Once daily, tablets of 3.2-4.8 mg of dried Vitex fruit extract were utilized. Women's sexual function did not differ between the intervention's beginning and end for 4 wk (p > 0.05) (6).

#### 3.2.1.2. Red clover (*Trifolium pratense*)

*Trifolium pratense*, is a plant containing phytoestrogens. Use of red clover as a 2% vaginal cream once a day for 8 wk was studied to see how it affects the postmenopausal women's sexual dysfunction. Postmenopausal women who took red clover experienced improved sexual function (p = 0.001) (13). Red clover has been shown to improve performance and sexual health in menopausal women as well. Compared to the control group, menopausal women who took 2 red clover 40 mg capsules daily for 90 days had these benefits, and a reduction of vaginal dryness (47% vs. 83%), dyspareunia (34% vs. 69%), and decreased libido (52% vs. 73%), were observed (14).

#### 3.2.1.3. *G. biloba*

Flavonol glycosides account for 24% of the phytoestrogen-rich *G. biloba* plant extract. *G. biloba* improves blood flow, modifies nitric oxide systems, and relaxes soft muscular tissues, which can impact the sexual response cycle (15). The effect of *G. biloba* extract capsules on postmenopausal women, aged between 50-60, who were sexually active were examined. After taking 2-4 tablets daily for one month, or 120-240 mg (each containing 60 mg of *G. biloba* extract), postmenopausal women did not experience any statistically significant differences in their sexual activity compared to the placebo group. The intervention, however, increased sexual desire (p = 0.02) (16). The effect of *G. biloba* capsules on postmenopausal women's sexual satisfaction was not observed in another study (15). The impact of *G. biloba* pills and aromatherapy inhalation components on the sexual function of postmenopausal women was examined. In the *G. biloba* group, 40 mg pills were taken 3 times per day. In contrast, in the aromatherapy group, 2-3 drops of an aromatherapy solution (lavender, fennel, rose, and geranium) were recommended each day for 6 wk. Sexual function in postmenopausal women was enhanced by aromatherapy and *G. biloba* supplements (p < 0.001) (17).

#### 3.2.1.4. *Fennel*

*Foeniculum vulgare*, a carrot family member, is a native of the Mediterranean Sea coast. Iran is one of the nations where this plant is produced. The primary chemical constituents of fennel are trans- and dianethole, which have estrogenic actions. The impact of fennel vaginal cream on postmenopausal women's dyspareunia and sexual function were examined. An 8 wk prescription for a vaginal lotion containing 30 mg of 5% fennel, boosted sexual satisfaction and decreased dyspareunia (p < 0.001) (18). The impact of fennel vaginal cream on postmenopausal women's sexual function was researched. A fennel vaginal cream dose of 5 mg every night was utilized for this purpose for 8 wk. Women's sexual function was enhanced by the intervention (p < 0.001) (19).

#### 3.2.1.5. Squill

The squill bulb, ranging from 3-15 cm in diameter, has a pear-like shape and is found worldwide. The sea onion is also referred to by its scientific name, *Drimia maritima*. In squill extract, flavonoids have been found such as quercetin, which has phytoestrogen properties such as boosting libido and stimulating sexual desire (20). The effects of squill oil were investigated in postmenopausal women. During the $4^{\text{th}}$ wk, they applied squill oil on the clitoris in a dose of 3 ml 2-3 times each week, 5 min before sexual intercourse. When compared to the control group, the intervention group's levels of dyspareunia and sexual satisfaction were significantly higher (p < 0.001) (21). The impact of topical squill oil on the sexual function of reproductive-age women was examined. Squill oil was applied topically to the clitoris 1-3 times per week, 5-10 min before sexual intercourse for one month, squill oil enhances female sexual performance (p < 0.001) (20).

#### 3.2.1.6. Chamomile

Chamomile is the most frequently employed in medicinal use as Compositae or Asteraceae family herb. According to pharmacological studies, chamomile extract consists of apigenin and chrysin flavonoids which have phytoestrogens effects and can bind both classical and non-classical estrogen receptors to produce estrogenic effects. Postmenopausal women who used chamomile vaginal gel reported less painful sexual intercourse and greater sexual satisfaction (p < 0.001) (12).

#### 3.2.1.7. Ginseng

According to C.A. Meyer, Korean red ginseng (KRG), also known as *P. ginseng* root, has been utilized as a traditional herbal remedy in Asian nations for a very long time. Ginseng is a potent phytoestrogen with excellent antioxidant effects (22). Red ginseng's impact on 41 reproductive-age women was studied by double-blind, cross-over clinical trial; the intervention group received 1 gr of ginseng capsules 3 times per day for 8 wk. In the group receiving ginseng, the intervention increased sexual desire, arousal, orgasm, and sexual satisfaction. However, no significant difference was observed in sexual function between the intervention and control groups (p = 0.07) (23). The impact of *P. ginseng* on postmenopausal women's sexual function was examined in another study, 500 mg of *P. ginseng* was administered twice a day for 4 wk, which improved women's sexual performance in comparison to the control group (p < 0.001) (24). The impact of KRG on the sexual arousal of 32 postmenopausal women was examined. Taking 1 mg ginseng in the form of pills 3 times per day for 8 wk increased sexual arousal (p = 0.006) (25).

#### 3.2.1.8. *T. foenum-graecum* or Fenugreek

This plant, which belongs to the family of legumes, contains phytoestrogens. The progesterone-like actions of the sapogenin are found in *T. foenum* extract. Diosgenin, a substance with estrogenic and steroid hormone-like properties, is also present in this extract. The effect of *T. foenum-graecum* seed extract (Libifem) on testosterone, estradiol and sexual function in healthy menstruating women was examined, this clinical trial's findings showed that healthy menstrual women who self-reported impaired sexual function responded well to 600 mg/day of a specialized extract of *T. foenum-graecum* seed extract (Libifem). Significant improvements were seen in both arousal and sexual desires (p < 0.001) (26). Fenugreek vaginal cream's impact on postmenopausal women's dyspareunia and sexual satisfaction was studied (27).

#### 3.2.1.9. *Apium graveolens L.* fruit (celery seed)

The plant known as “celery" is *Apium graveolens L*. One of the plants rich in flavonoids including apigenin, is celery. Celery seed includes phytoestrogens components that may assist in the lubrication and vasocongestion of the clitoral and vaginal canals, which enhance the sexual function of females. Celery seed's impact on female sexual dysfunction was studied, the administration of celery seed for 6 wk could considerably enhance female sexual function by improving sexual desire, arousal, lubrication, and pain relief during intercourse. Those receiving celery seed had significantly higher, overall, FSFI scores as compared to those receiving a placebo (p < 0.001) (28).

#### 3.2.1.10. Carrot seed (*Daucus carota*)

The carrot is one of the plants emphasized in traditional Iranian medicine. Carrot seeds have been described as a contraceptive, aphrodisiac, emmenagogue, and antifertility agent in some European literature (29).

The impact of carrot seeds on lowering libido in women of reproductive age was examined. In this study, 500 mg carrot seeds were given 3 times per day for 12 wk. The intervention enhanced sexual performance (p < 0.001) (30).

The impact of the Jazar supplement on postmenopausal women's quality of life and sexual performance was investigated, 4 Jazar capsules (500 mg each capsule) which contains Vitex, fennel, and carrot seeds, were recommended daily. Postmenopausal women's sexual function was enhanced by the intervention (p < 0.001) (29).

#### 3.2.1.11. Pomegranate (*P. granatum L.*)

Several qualities of *P. granatum L*. ellagitannins which can be astringent or possess traits connected to protein molecules, are one of the principal and significant chemicals in pomegranate bark that ultimately induce muscles to stretch and contract. In addition, phytoestrogen is present in pomegranate bark, and its gel affects is found to affect orgasm and sexual satisfaction in women of reproductive age. 3% pomegranate bark gel was applied 3 times weekly, 15 min before sexual contact for consecutive 8 wk. Sexual satisfaction and orgasm were enhanced in the intervention group in comparison to the control group (p < 0.001) (31).

#### 3.2.1.12. Maca

Maca is the root of the Lepidium meyenii plant, which grows exclusively at high altitudes in the Andes region of Peru where it is widely used for its putative fertility-enhancing and aphrodisiac properties. In a study, menopausal women who consumed 3.5 mg of mace powder daily for 6 wk had their sexual performance examined. Using Greene Climacteric Scale questionnaire, they discovered that sexual issues drastically diminished following intake (p = 0.05) (32).

#### Non-phytoestrogens plants

#### 3.2.2.1. *M. officinalis* (Lemon balm)

A tonic for the major organs, *M. officinalis*, often known as lemon balm (Labiate), is an aromatic and mildly peppery plant with a lemon flavor and aroma. In a study, lemon balm affected sexual dysfunction in women between 18 and 50. 500 mg capsules of lemon balm extract were given twice a day for 4 wk (p < 0.001) (33).

#### 3.2.2.2. Aphrodite

The herbal supplement Aphrodite contains Zygophyllacea, ginger, saffron, and cinnamon, each with unique medicinal benefits. Aphrodite's impact on orgasm and sexual desire in postmenopausal women was examined in a study. 2 Aphrodite capsules daily for one month were given to the postmenopausal women in the intervention group, which increased libido and orgasm compared to the control group (p = 0.02). Aphrodite capsules contain 40 mg of Zygophyllaceae fruit, 27.12 mg of ginger, 3.3 mg of saffron, and 11 mg of cinnamon (34). Aphrodite intake increased menopausal women's sexual satisfaction, according to a different study (p = 0.01) (35).

#### 3.2.2.3. Date palm pollen

A member of the Palmaceae family of plants, the palm tree (*Phoenix dactylifera*) has various medicinal uses. Date palm pollen contains fatty acids, antioxidants, vitamins A and E, as well as a wealth of minerals, according to phytochemical studies. Date pollen capsules' impact on postmenopausal women's vaginal dryness and dyspareunia was studied. Date pollen capsules containing 300 gr of the substance were recommended for 35 days. Vaginal wetness and dyspareunia significantly differed between the 2 groups (p = 0.001 and p = 0.048, respectively) (36). The impact of date pollen capsules on orgasm and sexual satisfaction in postmenopausal women was examined. For 35 days, postmenopausal women took 300 mg of date pollen capsules, which increased orgasm in the intervention group compared to the control group (p = 0.004) but did not affect the women's sexual satisfaction (37).

#### 3.2.2.4. *Althaea Officinalis* or Alcea

This plant is a type of medicinal plant that contains a lot of mucilage and moisturizing properties. The impact of Alcea on postmenopausal women's sexual function was examined. 5% Alcea vaginal suppository was administered each night for the first 2 wk and every other night for the following 6 wk. The intervention had a favorable impact on postmenopausal women's sexual function compared to the placebo group (p = 0.001) (38).

#### 3.2.2.5. *Tribulus terrestris L.* (Zygophyllaceae) 

*Tribulus terrestris L*. (Zygophyllaceae) is native to India. Protodioscin a steroidal saponin found in Tribulus preparations, influences hormonal activity and the generation of endogenous androgen by causing a rise in the release of luteinizing hormone (39). The effect of hydroalcoholic extract of Zygophyllaceae in the form of syrup taking 0.9 mg twice a day for 8 wk was investigated on 60 postmenopausal women compared to 60 people in the control group. This intervention increased sexual satisfaction (p < 0.005) (40). The impact of Zygophyllaceae extract on 40 women of reproductive age was examined. Compared to the control group, sexual function increased when the section was administered in the form of 250 mg tablets 3 times per day for 120 days (p < 0.001) (41).

The effect of Zygophyllaceae extract was investigated on postmenopausal sexual disorders. 250 mg of extract was administered as treatment, 3 times daily for 120 days. Among the intervention group, sexual function had improved (p < 0.01) (42). The Zygophyllaceae was studied on 60 postmenopausal women as opposed to 30 individuals in the control group. For 90 days, the extract was recommended in 250 mg tablets to be taken thrice daily. The intervention enhanced sexual function (43). 7.5 ml of Zygophyllaceae extract was administered twice daily for one month to women of reproductive age. In the intervention group, Zygophyllaceae extract increased sexual function (p = 0.04) (44).

#### 3.2.2.6. Saffron

Because it contains substances like crocin and safranal, saffron has a stimulating and arousing influence on the senses. Saffron promotes sexual satisfaction and vaginal wetness in lab animals (45). Saffron's impact on sexual dysfunction in women between the ages of 18 and 39 was studied. In comparison to the control group, sexual function was improved by taking 15 mg saffron extract capsules twice daily for 8 wk (p < 0.001) (46). The sexual function of postmenopausal women was examined. The study aimed to investigate the effects of oral saffron in a daily capsule (containing 30 mg of dry saffron stigma powder) for 4 wk (p < 0.001) (45).

**Figure 4:**
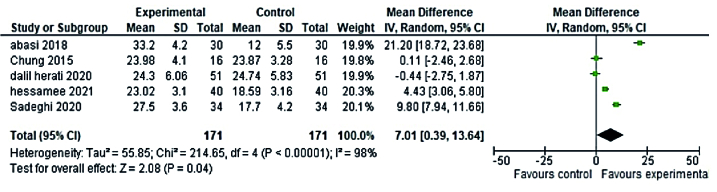
Forest plot of the phytoestrogens plants and sexual health of premenopausal women.

**Figure 5:**
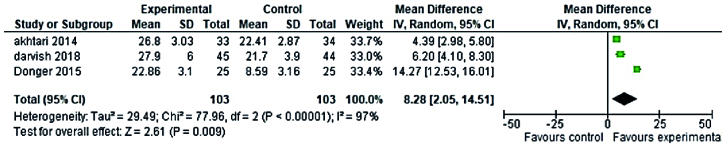
Forest plot of the non-phytoestrogens plants and sexual health of premenopausal women.

**Figure 6:**
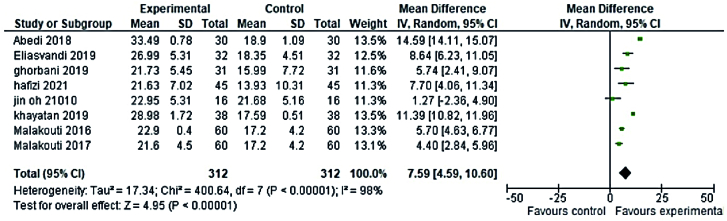
Forest plot of the phytoestrogens plants and sexual health of postmenopausal women.

**Figure 7:**
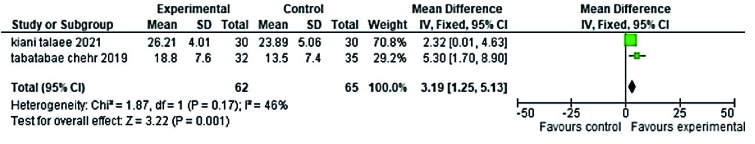
Forest plot of the non-phytoestrogens plants and sexual health of postmenopausal women.

Randomized clinical trial (RCT) studies of phytoestrogens plants (5 RCTs studies in the reproductive period and 8 RCTs studies in the menopause period) and 5 RCTs studies of non-phytoestrogens plants (3 RCTs studies in the reproductive period and 2 RCTs studies in menopause period) that used the FSFI questionnaire and reported the mean and standard deviation were included in meta-analysis. No discernible difference was observed between phytoestrogens and non-phytoestrogens plants' effects on premenopausal women's sexual health, according to the meta-analysis (MD = 7.01; 95% CI = 0.39-13.64 and MD = 8.28; 95% CI = 2.05-14.5, respectively) (Figures 4, 5). The effects of phytoestrogen and non-phytoestrogen plants on the sexual health of postmenopausal women appear to differ significantly from one another (MD = 7.59; 95% CI = 4.56-10.60, and MD = 3.19; 95% CI = 1.25-5.13, respectively) (Figures 6, 7).

**Table 2:** Clinical trials conducted on herbal medicines effective on sexual health of premenopausal women


**Author, yr (Ref)**	**Variables**	**Study sample size**	**Intervention group/Group 1**	**Complication**	**Results**	**Tools**
**Dalil Heirati ** ***et al.*** **, 2020 (6)**	Sexual dysfunction in reproductive age women	102	Tablets containing Vitex agnus-castus once a day for 4 wk/51	Nausea	Effectless on sexual function (p > 0.05)	FSFI
**Ghavami ** ***et al.*** **, 2020 (47)**	Satisfaction with sex in sexually active women	60	2 times a week for a month, use herbal mixture (fenugreek seeds, pomegranate peel, and oak) as vaginal suppositories/30	No	Enhancing orgasm but having little impact on sexual satisfaction	Larson and FSFI
**Abbasi Pirouz ** ***et al.*** **, 2018 (20)**	Sexual function in women of reproductive age	60	5 min before sexual activity, use 1-3 drops of squill oil topically for one month/30	No	enhancing sexual performance	FSFI
**Darvish-Mofrad-Kashani ** ***et al.*** **, 2018 (33)**	Sexual dysfunction in women	89	500 mg capsules of *Melissa officinalis* (Lemon balm) extract twice a day for 4 wk/45	No	Improving sexual function (p < 0.001)	FSFI
**Mohammadzadeh ** ***et al.*** **, 2019 (31)**	Orgasm and sexual satisfaction	110	*Punica granatum* gel 3 times a week and 15 min before sexual contact with an applicator (vaginal) for 8 wk/55	No	Improving orgasm and sexual satisfaction (p < 0.001)	FSFI
**Hessami ** ***et al.*** **, 2021 (28)**	Women's sexual dysfunction	80	500 mg of celery seed 3 times each day for wk/40	No	Improving sexual function (p < 0.001)	FSFI
**Sadeghi ** ***et al.*** **, 2020 (30)**	Hypoactive sexual desire disorder in women of fertile age	68	500 mg of carrot seed 3 times per day for 12 wk	No	Improving sexual function (p < 0.001)	FSFI
**Vale ** ***et al.*** **, 2018 ${(41)}^{\text{a}}$ **	Women in premenopause with a disease of hypoactive sex desire	40	*Tribulus terrestris* 750 mg/day (3250 mg pills each day) for 120 days	_	Improving sexual function	FSFI
**Rahmati ** ***et al.*** **, 2017 (46)**	Sexual dysfunction in women of reproductive age	69	15 mg saffron extract tablet for 8 wk/35	No	Saffron improves women's sexual function (p < 0.001)	FSFI
**Donger ** ***et al.*** **, 2015 ${(48)}^{\text{b}}$ **	Sexual function in women	50	300 mg of a highly concentrated ashwagandha root extract twice daily for 8 wk/25	- Improving sexual function (p < 0.001)	FSFI
**Akhtari ** ***et al.*** **, 2014 (44)**	Sexual dysfunction in women	67	7.5 mg of *Tribulus terrestris* extract each day for 4 wk/33	Abdominal cramp	Improving sexual function	FSFI
**Chung ** ***et al.*** **, 2015 ${(23)}^{\text{c}}$ **	Sexual function in premenopausal women	41	3 ginseng capsules (1 g each) (crossover) for 8 wk/12 (KRG-placebo)	Mild gastric discomfort	KRG treatment significantly improved sexual desire, arousal, orgasm, and satisfaction domains	FSFI
**Rao ** ***et al.*** **, 2015 ${(26)}^{\text{d}}$ **	Sexual function and testosterone in healthy menstruating women	80	Libifem, 600 mg/day dosage of *Trigonella foenum-graecum* seed extract, was administered across 2 menstrual cycles /40	Headaches getting worse, indigestion/reflux	Sexual arousal and desire are rising	DISF-SR
All articles were done in Iran except a: Brazil, b: India, c: Korea, d: Australia. All control groups received placebo. FSFI: Female sexual function index, KRG: Korean red ginseng, DISF-SR: Derogatis sexual function interview						

**Table 3:** Clinical trials conducted on herbal medicines effective on sexual health of postmenopausal women


**Author, yr (Ref)**	**Variables**	**Study sample size**	**Intervention group/Group 1**	**Complication**	**Results**	**Tools**
**Hafizi ** ***et al.*** **, 2021 (29)**	Life satisfaction and sexual performance in postmenopausal women	90	4 Jazar capsules (500 mg each) daily for 8 wk/45	No	Improving sexual function (p < 0.001)	FSFI
**Khayatan ** ***et al.*** **, 2019 (13)**	Female postmenopausal sexual function	76	2% red clover vaginal cream once daily for 8 wk/38	No	Improving sexual function (p < 0.001)	FSFI
**Afshar ** ***et al.*** **, 2020 (49)**	Menopausal women in good health with sexual dysfunction	48	1000 mg pill of *Melissa officinalis*, fennel extract, and *Nigella sativa* powder once daily for 8 wk/27	No	Not improve the sexual function	FSFI
**Kianitalaei ** ***et al.*** **, 2021 (38)**	Female postmenopausal sexual function	60	Alcea vaginal suppository 5% every night for 2 wk. After that for 6 wk, use it every other night/30	Urinary frequency and dysuria	Improving sexual function (p < 0.001)	FSFI
**Najar ** ***et al.*** **, 2015 (18)**	Sexual satisfaction and dysparonia in postmenopausal women	60	5% fennel vaginal cream, 30 mg, for 8 wk/30	_	Reducing dyspareunia and increasing sexual satisfaction (p < 0.001)	Larsson questionnaire
**Mazalzadeh ** ***et al.*** **, 2018 (27)**	Postmenopausal women's dysparonia and sexual satisfaction	60	5% fenugreek vaginal cream application for 8 wk/30	_	Increasing sexual desire and decreasing dyspareunia (p < 0.001)	Larsson questionnaire
**Abedi ** ***et al.*** **, 2018 (19)**	Postmenopausal women's sexual function	60	5 gr of vaginal fennel cream each night for 8 wk/30	No	Improving sexual function (p < 0.001)	FSFI
**Ghorbani ** ***et al.*** **, 2019 (24)**	Menopausal women's sexual dysfunction	62	500 mg of *Panax ginseng* twice daily for 4 wk/29	Insomnia, palpitations, and flushing (ginseng group) gastric discomfort and change in urine color and smell (control group)	Improving sexual function (p < 0.001)	FSFI
**Malakouti ** ***et al.*** **, 2017 (17)**	Female sexual function in the postmenopausal phase	180	Tablets of *Ginkgo biloba* and a placebo aromatherapy group/60	Headach (*Ginkgo biloba* group) and forearm itching (aromatherapy group)	Improving sexual function (p < 0.001)	FSFI
**Taavoni ** ***et al.*** **, 2016 (34)**	Menopausal women's sexual desire and orgasm	80	Aphrodite capsules, which also include *Tribulus terrestris*, ginger, saffron, and cinnamon. Twice daily for 1 month/40	No	Improving sexual desire and orgasm	Sabbatsberg sexual function scale
**Taavoni ** ***et al.*** **, 2014 (35)**	Postmenopausal women's sexual satisfaction	80	Aphrodite capsules, which also include *Tribulus terrestris*, ginger, saffron, and cinnamon. Twice daily for 1 month/40	No	Improving sexual function (p = 0.01)	Sabbatsberg sexual function scale
**Amiri Pebdani ** ***et al.*** **, 2013 (15)**	50-60 yr-old postmenopausal women's sexual satisfaction	80	*Ginkgo biloba* extract, 120-240 mg every day for 30 days/40	No	Effectless on sexual satisfaction	Sabbatsberg sexual function scale
**Amiri Pebdani ** ***et al.*** **, 2013 (16)**	50-60 yr-old postmenopausal women's sexual activity	80	*Ginkgo biloba *extract, 120-240 mg every day for 30 days/40	No	Effectless on sexual activity	Sabbatsberg sexual function scale
**Yusefzadeh ** ***et al.*** **, 2017 (37)**	Menopausal women's orgasm and sexual satisfaction	60	35 days of date pollen capsules/30	No	Increasing orgasm (p = 0.004) but effectless on sexual satisfaction	FSFI
**Malakouti ** ***et al.*** **, 2016 (2)**	Female sexual function in the postmenopausal phase	120	Drops of *Ginkgo biloba* Jaroma solution 3 times each day on the skin of the forearm for 6 wk/60	Forearm itching of aromatherapy group	Improving sexual function (p < 0.001)	FSFI
**Tabatabaeichehr ** ***et al.*** **, 2020 (45)**	Postmenopausal women's sexual function	67	Take 1 saffron capsule every day for 4 wk/32	_	Improving sexual function (p < 0.001)	FSFI
**Tadayon ** ***et al.*** **, 2018** **(40)**	Satisfaction from sex in postmenopausal women	60	*Tribulus terrestris* extract syrup for 8 wk at a dosage of 0.9 mg/30	_	Improving sexual satisfaction (p < 0.005)	Larsson questionnaire
**de Souza ** ***et al.*** **, 2016 ${(42)}^{\text{a}}$ **	In postmenopausal women, a hypoactive sexual drive condition exists	36	Take 250 mg of *Tribulus terrestris* orally 3 times per day for 120 days/25	Nausea	Improving sexual function (p < 0.01)	FSFI
**Postigo ** ***et al.*** **, 2016** ** ${(43)}^{\text{a}}$ **	Menopausal women's sex functions	60	3 times per day for 90 days, take 250 mg Bindii (Tribulus) extract tablets/30	Diarrhea, nervousness, dizziness, and nausea (tribulus group) and nervousness, facial flushing, dizziness, and nausea (placebo group)	Improving sexual function	${\text{(SQ-F)}}^{2}$ and (FIEI) questionnaire
**Brooks ** ***et al.*** **, 2008 ${(32)}^{\text{b}}$ **	Postmenopausal women's psychological signs and tests for sexual dysfunction	40	3.5 gr of maca per day orally for 6 wk/20	No	Reduction of sexual dysfunction	GCS
**Oh ** ***et al.*** **, 2010 ${(25)}^{\text{c}}$ **	Menopausal women's sexual arousal	32	3 ginseng capsules for 8 wk, each containing 1 gr of ginseng/15	Vaginal bleeding	Enhancing sex arousal	FSFI
**Chedraui ** ***et al.*** **, 2006 ${(14)}^{\text{d}}$ **	Sexual and vaginal health after menopause	53	90 days of daily administration of 2 capsules of MF11RCE (80 mg of red clover isoflavones) or a placebo of comparable design. The medicine was used for another 90 days following a 7-day washout interval	No	Benefits for sexual and genital health	Researcher made scale
**Eliasvandi ** ***et al.*** **, 2019 (50)**	Female postmenopausal sexual function	64	Carnation, anise, anison, violets, *Terminalia chebula*, fresh green raisins, senna leaves, tangerine, and golqand were all included in herbal capsules taken twice daily for 4 wk/32	Abdominal pain	Enhancing sexual performance (p < 0.001)	FSFI
**Karimi ** ***et al.*** **, 2021 (21)**	In menopausal women, reducing dyspareunia and raising sexual satisfaction	60	2- 3 times a week, 5 min before sexual contact, 3 ml of squill oil on the clitoris and vaginal opening for 4 wk/30	No	Enhancing sexual satisfaction and decreasing dyspareunia (p < 0.001)	Sabbatsberg sexual self-rating scale and marinoff dyspareunia scale
**Bosak ** ***et al.*** **, 2020 (12)**	Sexual satisfaction and dyspareunia in postmenopausal women	96	5% chamomile vaginal gel, conjugated estrogen vaginal cream, and placebo gel were given to 3 groups (each with 32 participants) for 12 wk	Burning	Enhancing sexual satisfaction and decreasing dyspareunia (p < 0.001)	Larsson and a 4-degree pain self-assessment questionnaire
**Sadeghi Goghari ** ***et al.*** **, 2018 (36)**	Menopausal woman with vaginal lubrication and dyspareunia	65	35 days with 300 mg of date palm pollen /32	No	Enhancing lubrication of the vaginal canal and reducing dyspareunia	FSFI
All articles were done in Iran except a: Brazil, b: Australia c: Korea, and d: Santiago. All control groups received placebo. FSFI: Female sexual function index, SQ-F: Sexual quotient-female, FIEI: Female intervention efficacy index, GCS: Greene climacteric scale						

## 4. Discussion

In this systematic review and meta-analysis, the effects of medicinal plants that are phytoestrogens and non-phytoestrogens on the sexual health of pre- and postmenopausal women were compared. Several phytoestrogens and non-phytoestrogens medicinal plants are employed for women's sexual health, according to the findings of the current meta-analysis.

### The phytoestrogens medicinal plants

*P. ginseng* enhanced postmenopausal women's sexual function (24). KRG increased postmenopausal women's sexual arousal (25). Red ginseng's effect on women of reproductive age boosted libido, arousal, orgasm, and sexual pleasure (24). However, no discernible difference was observed between the intervention and control groups sexual function. In the rabbit clitoris, the corpora cavernosa and vaginal smooth muscle responded favorably to (KRG) excerpt. Castrated female mice exposed to KRG extract under in vivo circumstances, in the animal investigation, experienced an estrogenic response (23).

Red clover improved postmenopausal women's sexual function (13, 14). Applying squill oil topically to treat women's premenopausal and menopausal sexual issues proved successful. Quercetin, which produces phytoestrogen effects, including boosting libido and stimulating sexual desire, is the cause of these outcomes (20, 21).

*G. biloba* did not affect the activity or sexual pleasure of postmenopausal women, although it increased sexual desire (15). *G. biloba* improved the sexual function of postmenopausal women, in contrast to the another study, which is probably caused by the extended period of drug usage (6 wk in Malakouti's study and 4 wk in Amiri's study) (17). Fennel vaginal cream has a positive effect on dyspareunia and the sexual function of postmenopausal women (18, 19).

The sexual function of reproductive-age women improved after the consumption of carrot seeds (30). The Jazar supplement (including Vitex, fennel, and carrot seeds) was able to improve the sexual function of postmenopausal women (29). Using pomegranate bark gel improved orgasm and sexual satisfaction in women of reproductive age (31).

*T. foenum* vaginal cream increased libido and decreased dyspareunia in postmenopausal women (27). Similarly, another study on specialized *T. foenum-graecum* seed extract had a favorable impact on increasing sexual function, specifically on sexual desire and arousal in healthy menstrual women. This might result from the phytoestrogens this plant produces and the hormonal changes they cause (26). Using the Vitex plant increased women of reproductive age's orgasms, but their sexual function remained the same (6).

### The non-phytoestrogens medicinal plants

Lemon balm extract increased the desire and sexual satisfaction of reproductive-age women (33). *T. terrestris L.* (Bindii) was the subject of 5 studies that examined its impact on sexual health. The studies showed that *T. terrestris* improved women's postmenopausal and reproductive-age sexual function and sexual satisfaction (40-44). The active ingredients of *T. terrestris* can be metabolically transformed into weak androgens like dehydroepiandrosterone, which can then be transformed into stronger androgens like testosterone in the gonads and peripheral tissues, which are positively connected with sexual desire and sexual behavior (43).

Alcea improved menopausal women's sexual function. These results are probably brought on by this plant's moisturizing abilities (38).

Aphrodite was found to have a favorable impact on enhancing sexual function on orgasm, sexual desire, and sexual satisfaction in postmenopausal women (34). In studies examining the effect of saffron on sexual function before and after menopause, saffron was found to enhance sexual function (45, 46). 2 studies investigated the impact of date pollen capsules in postmenopausal women's vaginal moisture, dyspareunia, orgasm, and sexual satisfaction. The intervention increased vaginal moisture, dyspareunia, and orgasm but it had no effect on menopause women's sexual satisfaction, which can be influenced by some factors, including one's attitude toward aging, stress, anxiety, mental activity, and personal responsibility (36, 37). A systematic review and meta-analysis investigated the effects of phytoestrogens on sexual function in postmenopausal women. In this study, Soy had no effect on sexual function but it had improved the dyspareunia. Red ginseng did not significantly increase sexual performance, while maca and fenugreek significantly improved it (51). The previous study only examined medicinal plants that contained phytoestrogens, as opposed to the current study, which examined both phytoestrogen- and non-phytoestrogen containing plants' effects on the sexual health of women. A systematic review of medicinal herbs that affect menopausal women's sexual function and enjoyment was conducted, the results showed that Aphrodite, bindii, fennel, ginseng, fenugreek, and red clover all improved dyspareunia, sexual enjoyment, and sexual function. But date palm pollen and *G. biloba *did not affect sexual satisfaction (52). The authors of “A systematic review of herbal medicines to improve the sexual function of menopausal women" hypothesized that red ginseng, fennel, bindii, red clover, and black cohosh had the most significant impact on enhancing the menopausal women's sexual function (8). However, the current study looked at the effects of both phytoestrogens and non-phytoestrogen plants on the sexual health of both pre- and postmenopausal women.

These investigations looked at the effects of phytoestrogens and non-phytoestrogen plants on the sexual function of menopausal women. We discovered that these herbs benefit sexual function, although several medicinal plants, such as vitex, *G. biloba*, and ginseng, cannot be conclusively proven to have any effects. Therefore, further research with a larger sample size and a more focused technique is needed. Other findings of the present study are that there is no difference between phytoestrogens and non-phytoestrogens medicinal plants on the sexual health of premenopausal women, till, it seems that phytoestrogens medicinal plants are more effective in improving the sexual function of postmenopausal women than non-phytoestrogens medicinal plants.

The reason phytoestrogens are more effective than non-phytoestrogens in postmenopausal women is due to the low estrogen level in postmenopausal women compared to women of reproductive age. Phytoestrogens, weak estrogen agonists, can provide more substantial estrogenic effects when the amount of estrogen in the environment is low. Therefore, maybe this prediction is correct that they provide more estrogenic properties in postmenopausal women (53). On the other hand, the vasomotor symptoms of menopause, which hurt sexual function, are resolved by taking phytoestrogen. The elimination of these symptoms with the synergistic effect of phytoestrogen in increasing vaginal moisture has improved sexual function in postmenopausal women, but this is not true in women of reproductive age (6).

## 5. Conclusion

There is no difference between the effects of phytoestrogens and non-phytoestrogens medicinal plants in improving the sexual health of premenopausal women. But the effect of phytoestrogens medicinal plants is more in menopausal women, so using phytoestrogens medicinal plants such as fennel, fenugreek, and red clover with precaution (avoid high dose consumption) is a suitable choice for improving the sexual health of menopausal women.

##  Conflict of Interest

The authors declare that there is no conflict of interest.
